# The use of a synthetic shoulder patch for large and massive rotator cuff tears – a feasibility study

**DOI:** 10.1186/s12891-020-03227-z

**Published:** 2020-04-07

**Authors:** P. Cowling, R. Hackney, B. Dube, A. J. Grainger, J. D. Biglands, M. Stanley, D. Song, P. G. Conaghan, S. R. Kingsbury

**Affiliations:** 1 Leeds Teaching Hospitals NHS Trust, Chapel Allerton Hospital, Chapeltown Road, Leeds, LS7 4SA UK; 2 NIHR Leeds Biomedical Research Centre, Leeds, UK; 3 Leeds Institute of Rheumatic and Musculoskeletal Medicine, University of Leeds, Leeds, UK; 4 Leeds Medical School, University of Leeds, Leeds, UK

**Keywords:** Shoulder, Rotator cuff, Rotator cuff tear, Rotator cuff repair, Surgery, Patch, Augmentation

## Abstract

**Background:**

The aim of this study was to explore the feasibility of using a non-absorbable biocompatible polyester patch to augment open repair of massive rotator cuff tears (Patch group) and compare outcomes with other treatment options (Non-patch group).

**Methods:**

Participants referred to orthopaedic clinics for rotator cuff surgery were recruited. Choice of intervention (Patch or Non-patch) was based on patient preference and intra-operative findings. Oxford Shoulder Score (OSS), Shoulder Pain and Disability Index (SPADI), and Constant score were completed at baseline and 6 months. Shoulder MRI was performed at baseline and 6 months to assess fat fraction and Goutallier classification pre- and post- treatment. Feasibility outcomes (including retention, consent and missing data) were assessed.

**Results:**

Sixty-eight participants (29 in the Patch group, 39 in Non-patch group) were included (mean age 65.3 years). Conversion to consent (92.6%), missing data (0% at baseline), and attrition rate (16%) were deemed successful feasibility endpoints. There was significant improvement in the Patch group compared to Non-patch at 6 months in OSS (difference in medians 9.76 (95% CI 2.25, 17.29) and SPADI: 22.97 (95% CI 3.02, 42.92), with no substantive differences in Constant score. The patch group had a higher proportion of participants improving greater than MCID for OSS (78% vs 62%) and SPADI (63% vs 50%) respectively. Analysis of the 48 paired MRIs demonstrated a slight increase in the fat fraction for supraspinatus (53 to 55%), and infraspinatus (26 to 29%) at 6 months. These differences were similar and in the same direction when the participants were analysed by treatment group. The Goutallier score remained the same or worsened one grade in both groups equally.

**Conclusions:**

This study indicates that a definitive clinical trial investigating the use of a non-absorbable patch to augment repair of massive rotator cuff tears is feasible. In such patients, the patch has the potential to improve shoulder symptoms at 6 months.

**Trial registration:**

ISRCTN, ISRCTN79844053, Registered 15th October 2014 (retrospectively registered).

## Background

In the United Kingdom, 2.4% of all primary care consultations involve shoulder problems, and of these around 70% have rotator cuff pathology affecting their daily living [1, 2]. The prevalence of massive cuff tears ranges from 6.4–12% [3–5] with the management of symptomatic large and massive rotator cuff tears (RoCTs) presenting significant problems to the shoulder surgeon [6].

Repair of symptomatic RoCTs results in better clinical results compared to conservatively managed tears [7, 8]. Rates of re-rupture following repair vary significantly (7–46%) [9, 10] but for massive tears can be as high as 94% [8]. Commonly reported risk factors for failure are poor tendon quality, fatty atrophy, number of tendons involved, pre-operative tear size, tension on repair and patient age [11–14].

The management of large and massive RoCTs in shoulders with minimal arthritis remains a dilemma for shoulder surgeons [15, 16] . There are many options available, including specialised rehabilitation, simple arthroscopic surgery including debridement [17–20], long head of biceps tenotomy, bursectomy [21, 22], various tendon transfers [23, 24] through to formal repair of the tendon. This may be in the form of partial repair or complete repair where achievable. Current published outcomes for all these measures have been relatively successful, but re-tear rates are often high, and clinical outcomes can deteriorate after 2 years [25, 26]. This has therefore led to the development of further techniques, including use of patch to augment repair, subacromial spacer insertion and superior capsular reconstruction [27].

Surgeons have also investigated the use of patch grafts to bridge or augment larger tears in poor quality tendons with promising results [28–30]. However, these reports are predominantly case series with no attempt to compare the results with other, sometimes simpler, surgical options which may provide satisfactory outcomes [21, 31, 32]. The James Lind Alliance have also highlighted the need to examine different management options for repair of rotator cuff tears as a priority area of research [33].

We conducted a study to determine the feasibility of conducting a randomised controlled trial using patch-assisted surgery; and to compare the clinical outcomes and MRI findings of patients with massive RoCTs who were managed with patch-assisted surgery against patients managed with traditional options (non-surgical physiotherapy-based rehabilitation, arthroscopic repair, and arthroscopic debridement).

## Methods

### Design overview

The Shoulder Patch for Rotator Cuff (SPARC) study was a pragmatic, two-arm feasibility study. The study protocol was approved by Leeds West Research Ethics Committee (13/YH/0030) and registered on ISRCTN (ISRCTN79844053). Written informed consent was obtained for all participants prior to screening. Participants were recruited from 1st September 2013 to 20th October 2014. For the purposes of this feasibility study participants were followed up for 6 months (follow-up completed 20th October 2015).

### Setting and participants

Participants were recruited through the Leeds Teaching Hospitals NHS Trust orthopaedic clinics upon referral for rotator cuff surgery. Participants were eligible if they had ultrasound or magnetic resonance imaging (MRI) findings confirming massive rotator cuff full thickness tears (RoCT) (> 5 cm in size), with unacceptable pain and disability following conservative treatment or previous surgery that had failed and would be considered for patch surgery under routine clinical practice. Exclusion criteria were age < 18 years old; history of infection in the shoulder; neurological condition affecting the shoulder girdle; presence of rotator cuff arthropathy (secondary osteoarthritis of the glenohumeral joint as a result of a rotator cuff tear); current treatment for malignancy; pregnancy or lactation; currently participating in other research studies; or inability to give informed consent. Participants with contraindications to MRI were included but did not take part in the MRI component of the study.

### Treatment allocation and interventions

**Fig. 1:**
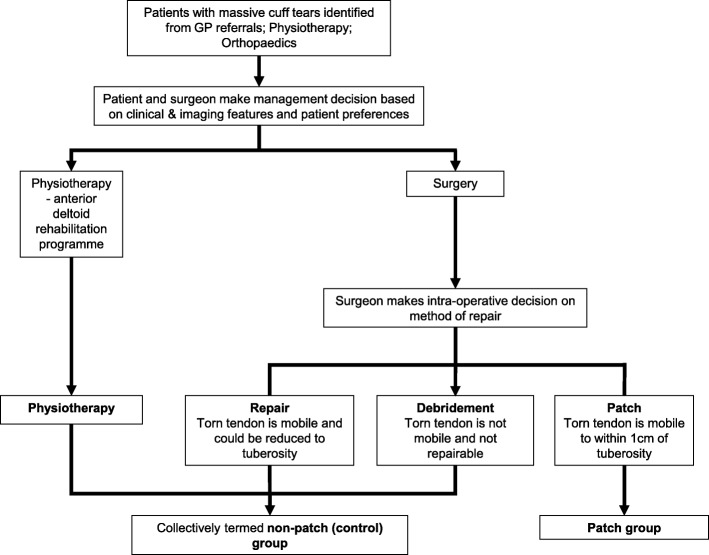
Treatment allocation flowchart. Intervention was determined in consultation between the surgeon and the participant. Management decisions were made at two stages. First, the surgeon and participant jointly made a decision between either conservative management (the anterior deltoid rehabilitation programme) or surgery. Second, an intraoperative decision on surgical management was made by the surgeon: if the torn tendon was mobile and could be pulled back to the tuberosity, arthroscopic repair was performed; if the torn tendon could be mobilised following release enough to allow attachment of a patch, repair was performed using a patch; and if the torn tendon could not be mobilized following release, a debridement was performed

The particular intervention was determined in consultation between the surgeon and the participant (Fig. 1 and Additional file 1: Table 1). Management decisions were made at two stages. First, the surgeon and participant jointly made a decision between either conservative management (the anterior deltoid rehabilitation programme) or surgery. Second, an intraoperative decision on surgical management was made by the surgeon. If the torn tendon was mobile and could be pulled back to the tuberosity, arthroscopic repair was performed; if the torn tendon could be mobilised following release to within 1 cm of the tuberosity to allow attachment of a patch, repair was performed using a patch; and if the torn tendon could not be mobilised following release, a debridement was performed. In summary, participants in the ‘Patch’ group had elected surgical management and had massive cuff tears which could not be fully reduced at surgery but were suitable for repair by patch augmentation. The ‘Non-patch’ group had massive tears and elected for either non-surgical rehabilitation, or surgical intervention involving a procedure other than a patch.

**Table 1:** Demographic and baseline characteristics

Characteristic	Patch (*n* = 29)	Non-patch (*n* = 39)	*P*-value
Age, years, mean ± SD	65.17 ± 8.98	65.38 ± 9.44	0.93
Sex, male	13 (46)	13 (33)	0.28
BMI, kg/m2, median (IQR)	29.71 (26.17–31.24)	26.75 (24.74–31.49)	0.69
Smoking status			
Current smoker	3/28 (11)	2/39 (5)	
Previous smoker	13/28 (46)	20/39 (51)	
Never smoked	12/28 (43)	17/39 (44)	
Cigarettes per day, median (IQR)	10 (5–20)	10 (10–20)	0.80
Employment history			
Employed	8/28 (29)	7/39 (18)	
Self-employed	1/28 (4)	4/39 (10)	
Unemployed	n/a	5/39 (13)	
Retired	19/28 (67)	23/39 (59)	0.13
Job activity (current/previous)			
Heavy manual	7/14 (50)	13/39 (48)	
Desk based	3/14 (21)	8/39 (30)	
Combined	4/14 (29)	6/39 (22)	0.71
Medical history			
Duration of symptoms, median (IQR)	11.5 (6–20)	12 (6–24)	0.64
Previous physiotherapy	19/28 (68)	29 (74)	0.56
Previous IA injection	9/27 (33)	12/36 (33)	0.99
Previous operation	4/28 (14)	7 (18)	0.48
Oxford score,median (IQR)	28.00 (21–34)	25.00 (20–30)	0.24
SPADI,median (IQR)	46.92 (30.77–71.54)	54.61 (42.31–66.92)	0.56
EQ-5D- Index, median (IQR)	0.70 (0.53–0.82)	0.72 (0.55–0.80)	0.87
Eq-5D- VAS, mean (SD)	70.6 (17.7)	74.0 (18.0)	0.43
Constant score,median (IQR)	44.00 (27–54.5)	42.00 (33.5–51.92)	0.75

Values are N (%) unless stated

Results from t-test, Mann-Whitney, chi-square test and Fisher’s exact test where appropriate

### Patch properties

The Leeds Kuff Patch (Xiros, Leeds UK) is produced from polyethylene terephthalate (PET, polyester), a non-absorbable biocompatible material that has been in use for the reconstruction of ligaments and tendons for over 25 years. Early generations of artificial tissue contained polytetrafluorethylene and polypropylene, which resulted in inflammatory reactions within the surrounding tissues [34] . However, more modern techniques and materials promote new tissue ingrowth and minimize the occurrence of a foreign body reaction [35].

The design of the patch comprises a base component with an integral reinforcement component. This base component has an “open structure” that acts as a scaffold allowing tissue ingrowth. The polyester is rendered hydrophilic during the manufacturing process while the reinforcement provides enhanced strength for the patch.

### Outcome measures and follow-up

Participants were followed up for 6 months with data collected using standardised case report forms at baseline (pre-treatment), 6 weeks and 6 months post-treatment. At all visits, clinical evaluations were performed. Baseline clinical data collection included age, gender, body mass index, duration of symptoms, range of movement, previous treatments for shoulder (including surgery), details of all therapies (including use of intraarticular therapies) in the previous 12 months. Participants completed a series of patient reported questionnaires at each visit to assess pain, function and patient satisfaction (Oxford Shoulder Score (OSS) [36], Shoulder Pain and Disability Index (SPADI) [37], and the Constant Score [38] and quality of life was assessed using the EuroQol five dimensions questionnaire (EQ-5D-5 L) [39]. An increase in the value of the OSS and Constant score at follow-up represents improvement, while for SPADI, a decreased score represents improvement in pain and function. Participants with no MRI contraindications had an MRI scan at baseline and 6 months.

### MRI scans

**Fig. 2:**
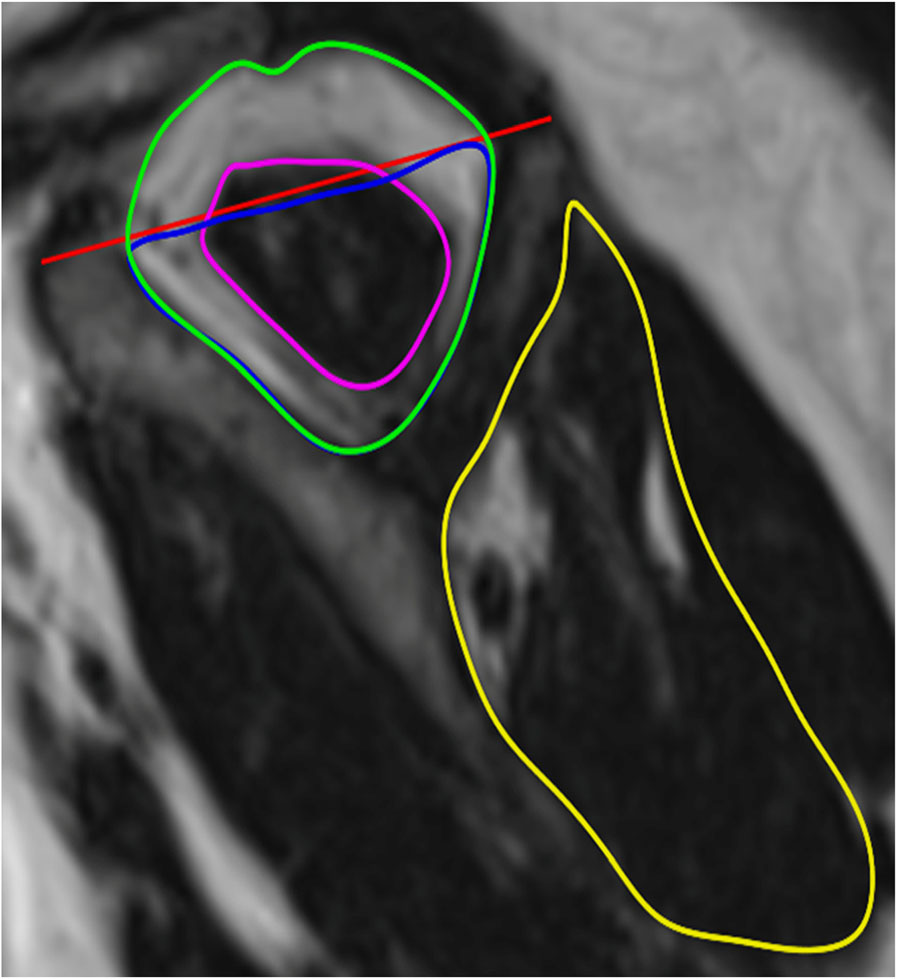
Areas contoured on OSIRIX. Green – Supraspinatus (SSP), Blue – Supraspinatus fossa (SSP_T), Pink – supraspinatus remnant (SSP_RM), Yellow – infraspinatus (ISP), Red – tangent line with normal TS

MRI scans of the shoulder were performed using a pre-agreed protocol using a MAGNETOM Verio 3.0 T MRI system (Siemens Healthcare, Erlangen, Germany). Fat and water images were generated from the Dixon images using the scanner vendor’s software. B0 variations due to changes in magnetic susceptibility at tissue interfaces were corrected for using a phase correction method [40]. Imaging analysis was performed using the OSIRIX system (Geneva, Switzerland). Images were contoured on the fat only images from the spinoglenoid notch medially to the first slice showing continuous bone marrow in the scapula laterally. This established a reproducible volume in patients, which did not suffer from signal drop off as previously described [41]. Regions of interest (ROIs) were drawn for the purpose of measuring fat fraction values. The 3 ROIs drawn outlined the supraspinatus fossa, bounded superiorly by the trapezius muscle (SSP), the suprapinatus remnant (SSP_RM) (defined as the muscle remnant within the SSP) and the infraspinatus muscle (Fig. 2). Fat fraction values were calculated for each ROI as the mean voxel value in the fat image ROI divided by the sum of the mean voxels values in the fat and the water images ROIs (fat/(fat+water)). Contouring was undertaken using the methods adopted by Zanetti et al. [41] by a single reader blinded to treatment allocation.

Muscles were also graded with the Goutallier Classification [42] using the 0–4 grading system.

### Sample size

As no hypothesis testing was anticipated no formal power calculations were performed. Published evidence for pilot study sample size suggests that 30 patients per group represent a good balance between accuracy of effect size estimation and feasibility [43–46]. Therefore we aimed to recruit 60 patients in total. Early in the study the protocol was amended to enable over-recruitment in order to allow for the recruitment of participants who met study criteria but were ineligible for MRI whilst still ensuring a total of approximately 60 participants took part in the MRI component of the study.

### Statistical analysis

Statistical analysis was conducted using STATA software, version 13 (College Station, TX, USA 2013). To understand feasibility, methodological issues relevant to this study were reported according to the framework devised for feasibility studies by *Shanyinde* et al. [47, 48]. Recruitment and eligibility was assessed using rate of eligibility (%), the conversion of eligible to consent (%) and an assessment of whether required numbers sought were recruited. Adherence to the protocol was assessed qualitatively by broadly reviewing the study objectives against our findings, and the acceptability assessed using the attrition rate (%) [49]. The outcome data was assessed based on the amount of missing data (%) found in each case report form.

The patch and non-patch groups were compared descriptively at baseline. At 6 month follow-up, differences between the medians in each group and associated 95% confidence intervals were assessed using quantile regression, adjusting for baseline scores. The groups were also compared for the proportions in each group that had improvement greater than the previously-reported minimum clinically important difference (MCID) in each shoulder-specific outcome measure [50]. This was expressed as percentages and odds ratios with 95% CI and adjusted for the baseline score for each outcome. MCID for OSS was set at 5 units, SPADI at 15.4 [51] and Constant score at 10.4 [52]. No hypothesis testing was undertaken as the study did not aim to draw inferences from the data.

#### Exploratory sub-group analysis

To further understand outcomes, sub-group analyses were performed. The patch group was further sub-divided into a ‘Patch-better Group’, where the surgeon subjectively rated the patch repair to involve relatively good quality tendon as noted at surgery (i.e. little delamination, thick tendon), and a ‘Patch-poor Group’ with massive tears retracted to the level of the glenoid with poor quality tendons (i.e. frayed, delaminated thinned tendon). The control group was also sub-divided into those who underwent an arthroscopic repair (Non-patch arthroscopy Group) and those who underwent simple arthroscopic surgery, including debridement (Non-patch excluding arthroscopy Group). Comparisons were made between i) the patch poor group and the patch better group and ii) the non-patch arthroscopy and the non-patch excluding arthroscopy using quantile regression as described above.

## Results

### Feasibility outcomes

#### Recruitment & eligibility

**Fig. 3:**
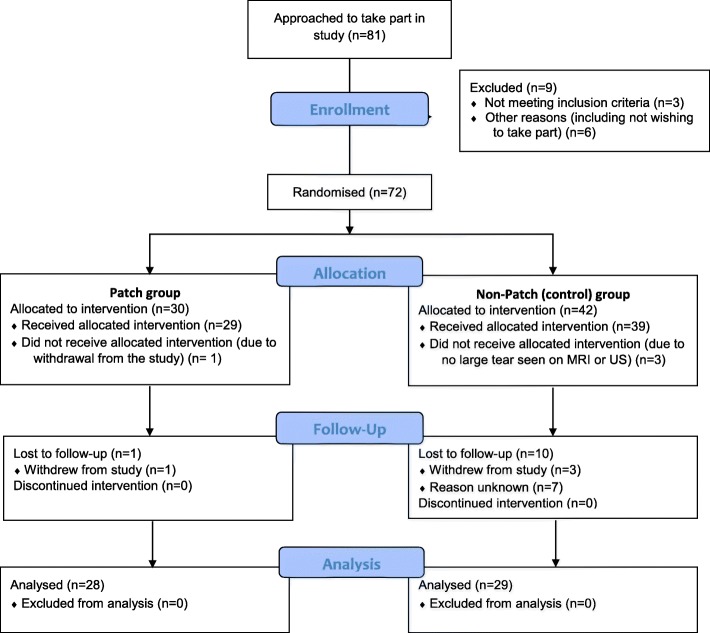
Participant flow diagram

A total of 81 patients were invited to participate, of whom 75 agreed to take part in the study (conversion to consent = 92.6%) and 72 met the eligibility criteria (rate of eligibility = 96.3%). Of the 72 recruited into the study four patients were withdrawn at baseline: 3 did not have a RoCT at baseline (2 on MRI and 1 on arthroscopy), and one patient asked to be withdrawn having undergone no surgical procedure. Therefore a total of 68 participants were allocated to an intervention arm: 29 to the patch group and 39 to the non-patch (control) group (Fig. 3).

Baseline characteristics were balanced across treatment arms (Table 1). Participants were on average (SD) 65.3 years old (9.18), 61% women, BMI of 28.8 (5.69) with symptom duration of a median 12 months.

#### Clinical outcome measures

There was a 100% completion rate for all questionnaires at baseline with respect to calculation of outcome scores, however 6 individuals (9%) (3 in each treatment arm) had missing data for the clinician-measured “range of motion” and “power” components of the Constant score (Table 2). At 6 months, completion rates to enable calculation of total scores across all questionnaires were high (90–97%), with slightly more missing data in the nonpatch group compared to the patch group.

**Table 2:** Summary of findings against 9 methodological issues for feasibility research

Methodological issues	Findings	Evidence
1. Did the feasibility /pilot study allow a sample size calculation for the main trial?	Achieved, estimates obtained suitable for sample size determination for a trial. Measures of dispersion obtained (median and IQR). Effect size measures (differences in medians between groups obtained) and absolute differences between patch and control groups.	Tables 1 and 2 provide the estimates obtained for different outcomes.
2. What factors influenced eligibility and what proportion of those approached were eligible?	No evidence of cuff tears and participant refusal.	Following the initial ultrasound and clinical examination which indicated presence of a massive cuff tear, 3 patients were found to be ineligible after MRI (2 patients) or arthroscopy (1 participant) found no evidence of a cuff tear. 1 participant asked to withdraw from the study prior to baseline.
3. Was recruitment successful?	Recruitment was successful. High recruitment due to cross-referrals from upper limb surgeons in the same unit.	Of the potential 75 participants identified, 72 were recruited.
4. Did eligible participants consent?	High conversion to consent	100% conversion rate (Fig.1) all 72 that consented were allocated to a study arm
5. Was the intervention acceptable to the participants?	Not directly assessed but the high numbers recruited suggest little difficulty.	68 (94%) out of a possible 72 entered the study after consenting
6. Were outcome assessments completed?	Completion rate was 100% for all questionnaires at baseline Questionnaire completion rates varied at 6 months.	Oxford score 6 month questionnaire (patch 27/28 and controls 26/29) completed SPADI 6 month questionnaire (patch 28/28 and controls 28/29) completed Constant score 6 month questionnaire (patch 28/29 and controls 26/29) completed.
7. Were outcomes measured those that were the most appropriate outcomes?	All questionnaires assessed main areas of interest (shoulder pain and function) and also patient quality of life	All participants completed all items at baseline
8. Was retention to the study good?	Once recruited retention was very good for the patch group but appeared lower in the control group	1 patch patient with no 6 month data and 10 control patients with no 6 month outcome data
9. Did all the components of the protocol work together?	The study was a success as most components worked well	Recruitment went smoothly, 68 recruited in total from an initial estimate of 60 required

Methodological issues based on *Shanyinde* et al. (28) and *Bugge* et al. (29)

#### Adherence & acceptability

The initial recruitment target of 60 participants (30 per arm) was achieved. Clinical data was collected using standardised outcome measures as set out in the protocol. The original protocol defined follow-up at 6 weeks and 6 months, however patients undergoing any form of surgery were struggling to perform the movement and strength elements of some of the outcome measures at 6 weeks. The 6 week follow-up visit was therefore stopped.

Overall attrition from the study at 6 months was moderate (16%), suggesting that the protocol was acceptable. A difference was observed in attrition between the patch and non-patch group (1 participant in the patch vs 10 in the non-patch group); half of the non-patch patients lost to follow-up were in the physiotherapy group. The mean follow up time from ‘date of procedure’ to 6 month follow-up was 227 days from an initial target of 180 days (6 months).

### Clinical outcomes

Improvement was seen at follow-up for all clinical outcomes in both patch and control groups, except for the EQ-VAS which was slightly reduced in the control group (Table 3). Overall, improvements in the patch group were of a greater magnitude than for the non-patch control group. The median score was higher in the patch group compared to controls for the OSS (baseline-adjusted difference 9.76, 95% CI 2.25, 17.29, *p* = 0.01) and lower for SPADI (difference 22.97, 95% CI 3.0, 42.92, *p* = 0.03) but no substantive differences were seen for the total Constant score (*p* = 0.59). Compared to the non-patch group, the patch group had a greater proportion of participants demonstrating improvement (change greater than MCID) in two of the shoulder-specific patient reported outcomes, although not statistically significant: 78% vs 62% for OSS (Odds ratio 2.94, 95% CI 0.78, 11.02,*p* = 0.11); 63% vs 50% for SPADI (Odds ratio 2.66,95% CI 0.72,9.78, *p* = 0.15) but had a lower proportion of participants demonstrating improvement for the Constant score (50% vs 62% [Odds ratio 0.57,95% CI 0.18,1.90,*p* = 0.35)]. Quality of life scores (EQ-5D Index and EQ-VAS) were slightly improved in the patch group compared to the non-patch group.

**Table 3:** Summary of outcome measurers by treatment group over 6 month follow up

Outcome measure	Patch group		Non-patch		*Difference in medians patch group vs non-patch, (95% CI)	*P*-value
	Baseline	6 months	Baseline	6 months		
Oxford score (/48)	28 (21–34)	42 (35–47)	25 (20–30)	34 (26–44)	9.7 (2.3,17.3)	0.01
SPADI	46.9 (30.8–71.5)	16.9 (6.9–28.5)	54.6 (42.3–66.9)	39.6 (8.9–53.5)	23.0 (3.0,42.9)	0.03
EQ-5D Index	0.70 (0.53–0.82)	0.89 (0.82–0.94)	0.72 (0.55–0.80)	0.78 (0.51–0.94)	0.11 (− 0.04,0.27)	0.15
EQ-5D VAS	75 (54–80)	80 (70–90)	75 (65–90)	70 (60–80)	10 (−0.7,20.7)	0.07
Constant score (CS) Total (/100)	44 (27–54.5)	57.8 (45.8–66.5)	42.0 (33.5–51.9)	55.5 (43.5–64)	3.0 (−8.1,14.2)	0.59
Pain CS score (/15)	8 (6.5–8.5)	2.5 (1.0–4.5)	8.5 (7–9)	2.5 (1.0–4.5)	0.00 (−2.0,2.0)	0.99
ADL CS score (/20)	8 (5–10)	16 (13–19)	8 (6–10)	10 (8–18)	6.0 (1.7,10.3)	0.01
ROM CS score (/40)	24 (14–32)	33 (27–39)	25 (18–30)	35 (24–40)	4.0 (−11.4,3.4)	0.28
Power CS score (/25)	4 (2–4)	6.0 (2–10)	4 (2–7)	6.0 (4–10)	1.3 (−5.0,2.4)	0.50

*Results from quantile regression adjusting for baseline score

*Values are median (IQR)

### Exploratory sub-group analysis

Results of the sub-analyses showed no substantive differences, all *p* > 0.05 (Table 4). All comparator groups showed improvement at 6-month follow up with slight variation in the magnitude between them. Compared to the patch-poor group, the patch-better group had better improvement in the Constant score (9.6 score difference between the groups) and less improvement in SPADI (2.4 score difference) having adjusted for their baseline scores; and no difference between the groups for OSS. The non-patch (excluding arthroscopy) group improved by a greater magnitude compared to the non-patch arthroscopy group in all 3 outcome scores although this was also not substantive.

**Table 4:** Sub-group comparisons between patch and non-patch groups over 6 month follow up

Group	Oxford score	SPADI	Constant score
**Patch poor**			
Baseline	24 (21–36)	51.5 (31.5–73.1)	34 (27–58)
6 months	42 (35–48)	16.2 (6.2–17.7)	53 (40.5–65.5)
**Patch better**			
Baseline	29 (21–34)	38.8 (29.9–71.5)	45.5 (24–54.5)
6 months	42 (40–46)	20 (11.5,28.5)	58.5 (55.5–66.5)
*Difference in medians (95% CI)	0.0 (−8.2,8.2)	2.4 (−21.6,26.3)	9.6 (−5.9,25.2)
P-value	0.99	0.84	0.21
**Non-patch arthroscopy**			
Baseline	23.5 (19.5–29)	61.9 (40.4–67.7)	44.3 (37.5–50.2)
6 months	29 (29–31)	42.3 (10.8,49.4)	49.5 (43.5–64)
**Non-patch (excluding arthroscopy)**			
Baseline	26 (21–30)	51.5 (42.3–61.5)	41.5 (31–53.1)
6 months	35.5 (25.5–45.5)	30 (6.9–53.5)	56.5 (41.5–63.5)
*Difference in medians (95% CI)	−5.6 (−9.5,20.7)	2.36 (−34.9,39.6)	2.83 (−14.4,20.1)
P-value	0.45	0.90	0.74

* Results at 6 months from quantile regression adjusting for baseline score

Values are median (IQR)

### MRI outcomes

Of the 68 patients eligible for the study, 58 underwent MRI at baseline. Of the patients who did not have a baseline MRI, eight had metallic implants contraindicated to MRI, while one suffered claustrophobia. Another patient attended the scan, but could not tolerate it so it was halted prematurely without sufficient images for analysis. At 6 month follow-up 54/58 underwent MRI, meaning 4 additional patients declining or not attending, alongside the still contraindicated 10 patients from baseline, totalling 14 who did not have the 6 month MRI. On analysis of the MRI scans, for 4 patients, the fat and water images could not be reconstructed from the “in” and “out of” phase images. One patient was imaged in the wrong plane: a technical error in MRI protocol. One patient subsequently withdrew from the study, so their data was not analysed. This resulted in 48 participants with paired MRI scores available: 22 in the Patch group, and 26 in the non-patch group. Complete paired Goutallier data was available for 41 participants.

In the full sample, there was only a slight increase in the fat fraction for supraspinatus (SSP; 53 to 55%) and the infraspinatus (ISP; 26 to 29%) while the supraspinatus remnant (SSP_RM) showed no change (Table 5). These differences were similar and in the same direction when the participants were analysed separately by treatment status (patch vs nonpatch).

**Table 5:** MRI findings

	Whole group (*N*=68)	Patch group (*N*=29)	Non-patch (*N*=39)
**Fat fraction volume (%)**			
Paired scan data available	48	22	26
Supraspinatus fat fraction (%)			
Baseline	53.7	55.0	51.7
6 months	55.0	56.0	54.2
Supraspinatus remnant (%)			
Baseline	23.5	24.1	22.9
6 months	24.4	25.8	23.1
Infraspinatus fat fraction (%)			
Baseline	25.9	25.4	26.3
6 months	29.1	28.9	29.2
**Goutallier classification, N (%)**			
Paired scan data available	41	18	23
Supraspinatus			
Improved	1 (2.4)	1 (5.6)	0 (0)
Worsened	11 (26.8)	6(27.8)	5 (21.7)
Remained the same	29 (70.7)	11 (61.1)	18 (78.3)
Infraspinatus			
Improved	2 (4.9)	1 (5.6)	1 (4.3)
Worsened	10 (24.4)	5 (27.8)	5 (21.7)
Remained the same	29 (70.7)	12 (66.7)	17 (74.0)
Teres minor			
Improved	3 (7.3)	2 (11.2)	1 (4.3)
Worsened	11 (26.8)	5 (27.8)	6 (26.1)
Remained the same	27 (65.9)	11 (61.1)	16 (69.6)

A change in Goutallier classification was defined as a difference of at least 1 grade. For the SSP, 11 participants worsened over time (5 in the controls and 6 in the patch), while 30 remained the same and 1 improved. Results were similar for ISP and teres minor.

## Discussion

The primary aim of this study was to determine the feasibility of conducting a randomised controlled trial using patch-assisted surgery. A definitive randomised control trial appears achievable in terms of recruitment, eligibility, acceptability and outcome measures. The secondary aim was to compare the clinical outcomes of patients with massive RoCTs who were managed with patch-assisted surgery against patients managed with traditional treatment options (non-surgical physiotherapy-based rehabilitation, arthroscopic repair, and arthroscopic debridement). Improvements in shoulder symptoms were found in both patch and control groups at 6 months, but a greater magnitude of improvement was observed in patients receiving patch repair.

To our knowledge this is the first study to compare synthetic patch repair with a number of common treatment modalities for large and massive rotator cuff tears, including conservative management. It is also novel to include MRI analysis of the rotator cuff before and after these treatment modalities. Previous studies investigating patch augmentation of large or massive rotator cuff repairs have mainly been case series [21, 31, 32] . Few studies comparing synthetic patch to other treatment methods for symptomatic rotator cuff tear have been reported. These generally involve similar patient numbers with massive RoCTs to the current study (ranging from 21 to 60 patients with patch repair), and include a comparison of the synthetic patch with no patch [53]; a comparison with biological collagen patch as well as standard repair group [54]; a comparison of patch plus bone marrow and arthroscopic cuff repair [55]; and patch Vs partial repair [56]. The synthetic patch demonstrated a lower retear rate, and improvement in various functional outcome measures and strength.

### Feasibility outcomes

This feasibility study was designed as a pragmatic study, and thus randomisation and blinding was not assessed. A future larger scale study would likely compare patch-assisted surgery with non-patch assisted surgery, removing the non-surgical component within this feasibility study. As such, we would envisage the use of a standardised intra-operative decision system for surgeons, which would determine whether a participant was eligible for patch-assisted surgery and participants would be randomised at this point into one of two surgical treatment arms. Although the surgeon and potentially the patient (as a result of patch surgery requiring a larger opening, and therefore a larger scar, than non-patch arthroscopic surgery) would be aware of the treatment allocation, an independent assessor could conduct subsequent follow-up visits to reduce potential bias.

The moderate attrition rate at 6 months (11 lost to follow-up) suggests that the protocol was acceptable for participants; however, we did make the decision to remove the 6 week follow-up from the protocol early in the study due to the difficulty of patients in completing functional tests so early after any form of surgery. Missing data was minimal possibly due to the use of a number of routinely used shoulder questionnaires that were easy to complete. There was a noticeably lower completion rate/retention in the non-patch group, with half of these having received non-surgical management. It is possible that having made the decision not to proceed with surgery that these patients became disengaged from involvement in a ‘surgical’ study and there may also have been disengagement from the surgical team since these patients were no longer under their clinical care.

Various scoring systems are quoted in literature for post-operative assessment of rotator cuff surgery. In this current study we selected the OSS and Constant score, since they were the most well validated and frequently used to determine outcomes of RoCTs [9, 57]. In addition, we included the SPADI which has been shown to have reasonable validity and, although devised primarily as a rheumatological outcome score [37, 58], has been used to assess outcomes of RoCTs in the past [59]. We found that the Constant score had the most missing data fields of the three scores, which may reflect the multi-domain nature of the tool with functional tests which are difficult for some RoCT patients to complete and a mixture of patient-reported and clinician-reported outcomes. A recent systematic review of the psychometric properties of patient-reported outcomes in patients with rotator cuff disease found good evidence in support of the measurement properties of SPADI, limited overall evidence for OSS and mixed evidence for the Constant score, with positive evidence for responsiveness and reliability but negative evidence for hypothesis testing [60]. A recent international consensus process involving patients, clinicians and researchers, has defined the core outcome domains for shoulder disorder trials as ‘pain’, ‘physical functioning’, ‘global assessment of treatment success’ and ‘health-related quality of life’ [61]. Work to define a core outcome set based on these domains for clinical trials of people with shoulder pain is ongoing. A future study would therefore incorporate recommendations from this work to ensure alignment with international standards.

The level of MRI acceptability and attendance was high, despite ten patients with contraindications to such scans. In addition, the data available from the scans was of reasonable reliability and accuracy, with only 7 of the 48 paired scans having missing data for analysis of fat fraction and/or Goutallier Classification, which was due to technical errors with the protocols set for those patients’ scans.

### Clinical outcomes

This study provides preliminary evidence that any standard shoulder intervention may provide improvement in outcome scores, but that patch repair provides greater improvement in all outcomes at 6 months. The differences between the patch group and controls were substantial (greater than MCID) for the Oxford Shoulder Score. On further subgroup analysis, no substantive differences were found between patch patients with good quality and poor quality tendons, although this finding may have been hampered by small numbers.

MRI outcomes at 6 months demonstrated little difference between the patch group and control group, with both groups demonstrating either similar or slightly worse (i.e. higher) fat fraction within the rotator cuff. It may be that 6 months is too early in the rehabilitation phase following any treatment method to note anatomical changes. To confirm our findings of clinically meaningful improvement using a patch, a definitive trial is needed.

## Limitations

Due to the pragmatic, unblinded nature of the study, a patient’s treatment wishes were taken into consideration, which is common practice with surgical interventions. In this setting this was a feasible and ethical way to conduct the study but may potentially lead to bias.

This pragmatic nature allows assessment of a number of treatment measures for massive RoCTs, but this can lead to a heterogenous comparison group: the non-patch group of 39 patients included those undergoing arthroscopic rotator cuff repair, arthroscopic debridement of an irreparable tear, and non-operative physiotherapy management. However, in terms of participant demographic and baseline characteristics there were no substantive differences between groups.

The inclusion criteria for the study allowed patients to be eligible if they had ultrasound or magnetic resonance imaging (MRI) findings confirming massive rotator cuff full thickness tears (RoCT) (> 5 cm in size), with unacceptable pain and disability following conservative treatment or previous surgery that had failed and would be considered for patch surgery under routine clinical practice. This does mean there is a heterogeneous sample for both groups, (patch and non-patch) with some patients having primary massive tears, and others retears of previously repaired tendons. In this feasibility study, this was noted but did not form part of the outcome analysis. This factor could influence outcome measures, and in any subsequent trial following this feasibility study, this should be analysed.

The subgroup analysis into tendon quality and degree of retraction is subjective, and due to lower numbers compared in each group there was reduced power in those analyses. However, these analyses highlight important factors that could be could be useful for eligibility and stratification purposes in subsequent trials.

Of the baseline number of 68 participants, only 58 underwent MRI assessment prior to any intervention:, 8 participants did not have an MRI at baseline due to metallic implants, 1 due to claustrophobia and another became claustrophobic during the first scan. Therefore, although patients had legitimate reasons for failure to complete MRI assessment, this led to a reduction in the number of scans possible to assess. For this reason, we amended the protocol to allow additional recruitment to the study to compensate for those ineligible for the MRI. At 6 months, a further 4 declined or failed to attend. The drop-out rate for those eligible for MRI assessment was acceptable, suggesting that the MRI was an acceptable component of the protocol, although over a longer follow-up it is possible that this number would decrease further.

The two point Dixon imaging technique employed did not correct for T2* effects, eddy currents, noise related bias or the spectral complexity of fat.

## Conclusion

Promising short-term results can be achieved using patch augmentation for massive RoCTs. This study suggests that a patch may be beneficial, and given the positive outcomes from this study, a larger trial to investigate patch surgery is feasible.

## Supplementary information

**Additional file 1 Table S1.** Description of surgical interventions

## Data Availability

The datasets used and/or analysed during the current study are available from the corresponding author on reasonable request.

## Abbreviations

BMI: Body mass index
CI: Confidence interval
EQ-5D-5 L: EuroQol five dimensions questionnaire
ISP: Infraspinatus
MCID: Minimum clinically important difference
MRI: Magnetic resonance imaging
OSS: Oxford Shoulder Score
PET: Polyethylene terephthalate
RoCT: Rotator cuff tear
SPADI: Shoulder Pain and Disability Index
SSP: Supraspinatus
SSP_RM: Supraspinatus remnant
TS: Tangent sign
UCLA: University of California at Los Angles
VAS: Visual analogue score
VIBE: Volumetric interpolated breath-hold examination
