# Supplementary material for: The use of a synthetic shoulder patch for large and massive rotator cuff tears – a feasibility study
Source: BMC Musculoskelet Disord. 2020 Apr 7;21:213. doi: 10.1186/s12891-020-03227-z (PMC7140555; doi:10.1186/s12891-020-03227-z)
Supplement: Supplementary file 1 — Additional file 1 Table S1. Description of surgical interventions [file 12891_2020_3227_MOESM1_ESM.docx]

| **Surgical intervention** | **Arthroscopic or open** | **Approach/portals** | **Procedure** | **Decision making** | **Fixation method** | **Rehabilitation** |
| --- | --- | --- | --- | --- | --- | --- |
| **Patch fixation** | Initially arthroscopic, then open | Posterior viewing, lateral working, then open deltoid split | Assessment of rotator cuff, mobilization using suction diathermy | If cuff tendon can be mobilized enough to accept patch augmentation | No 2 Ethibond* & No 2 Orthocord^£^ fixing Leeds Kuff patch to tendon, No 5 Ethibond* for per-osseous fixation of patch to tuberosity | Polysling 3 weeks. Pendular exercises & passive ROM to 90 deg FLE & ABD, 30 deg ER. Active assisted movements at 3weeks, Active movements at 6 weeks, strengthening at 8 weeks |
| **Rotator cuff repair** | Arthroscopic | Posterior viewing, and lateral working portals for access to the cuff | Assessment of rotator cuff, mobilization using suction diathermy | If cuff tendon could be mobilized enough to the footprint, and was of good quality, arthroscopic repair was performed | Double row anchor fixation using Twinfix^$^ and Quick T^$^ anchors. | Polysling for 4 weeks with passive ROM only. Subsequent graded return to function under care of physio |
| **Debridement** | Arthroscopic | Posterior viewing, lateral working | Assessment of rotator cuff, mobilization using suction diathermy | Whether to include LHB tenotomy +/- subacromial decompression | No fixation performed | Polysling until block wears off. Anterior deltoid rehabilitation |

**Key**: * = (Ethicon, Somerville, NJ, USA); £ = (DePuy Sythes, West Chester, PA, USA); $ = (Smith and Nephew, Andover, MA, USA); ROM = range of motion; deg = degrees; FLE = flexion; ABD = abduction; ER = external rotation; physio = physiotherapy; LHB = long head of biceps
